# Pharmacy Students’ Perceived Ability to Implement the Pharmacist’s Patient Care Process before and after a Redesigned Case Study Series in the United States

**DOI:** 10.3390/pharmacy12020051

**Published:** 2024-03-19

**Authors:** Amy Henneman, Samantha Axtell

**Affiliations:** 1 Thomas F. Frist College of Medicine, College of Pharmacy and Health Sciences, Belmont University, Nashville, TN 37212, USA; 2 Gregory School of Pharmacy, Palm Beach Atlantic University, West Palm Beach, FL 33401, USA

**Keywords:** Pharmacist’s Patient Care Process, case studies, problem-based learning, student perceptions

## Abstract

Since the incorporation of the Pharmacist’s Patient Care Process (PPCP) into the American Association of Colleges of Pharmacy standards, the process has been integrated in a variety of ways across curriculums. A two-semester case-based course was redesigned as a four-semester case-based course formally integrating the PPCP. Pharmacy students completing the original, two-semester course series and those completing the first two semesters of the redesigned course were given a voluntary survey to assess their perceived ability to integrate the PPCP into practice after the completion of each course. A total of 107 pharmacy students, 61 students completing the original series and 46 students completing two semesters of the redesigned series, completed the survey. The pharmacy students completing the redesigned, four-semester course series had a significantly higher perception of their ability to integrate the PPCP into patient care compared to the students completing the original series. This included the perceived ability to ask appropriate questions, identify medication-related problems (MRPs), document MRPs, and develop a plan for addressing MRPs. A significant difference was found in the students’ perceived ability to ask pertinent questions in an appropriate manner, identifying and documenting MRPs, managing and solving MRPs, and documenting patient interactions. No significant difference was identified regarding the students’ perceived ability to document the interaction or ensure that treatment-related plans were implemented. The students’ perception regarding the importance of asking pertinent questions, identifying MRPs, and documentation in patient care did not differ between the cohorts. The case-based course series redesigned from two to four semesters with formal integration of the PPCP resulted in an increase in the students’ perceptions of their ability to integrate the PPCP into patient care.

## 1. Introduction

The Joint Commission of Pharmacy Practitioners (JCPP) adopted the Pharmacist’s Patient Care Process (PPCP) in 2014, providing a uniform process for the delivery of patient care specifically for pharmacists [1]. The PPCP is a five-step process that has been described elsewhere [1]. In response to the adoption of the PPCP, the 2016 Accreditation Council for Pharmacy Education (ACPE) standard 10 stated that each pharmacy school’s curriculum should prepare “students to provide patient-centered collaborative care as described in the Pharmacists’ Patient Care Process model endorsed by the Joint Commission of Pharmacy Practitioners [2]”. There have been several studies describing the implementation of the PPCP in various courses within both the didactic and experiential curriculum for students completing Doctor of Pharmacy (PharmD) programs within the United States [3,4,5,6,7,8,9]. These studies have provided various levels of assessment regarding the implementation of the PPCP and have described its integration in a variety of courses; however, data regarding students’ perceived ability to implement the PPCP are lacking. The objective of this study was to determine if a redesigned problem-based learning case study series impacted PharmD students’ perceived preparedness to implement the PPCP. Given the PPCP is intended to provide a framework for delivering patient-centered care, it is important that PharmD students feel they have the ability to apply the PPCP upon entering advanced pharmacy practice experiences (APPEs) so that they will work to implement the process when engaging in patient care.

A two-semester problem-based case study course in the PharmD curriculum was redesigned to be a four-semester problem-based case study series as part of a four-year PharmD curriculum to foster critical thinking and more formally integrate the application of the PPCP longitudinally across didactic years. The original two-semester problem-based case study course took place during the second pharmacy year and consisted of patient cases in which PharmD students were asked to interview the patient and create a SOAP note for each case. The students would not have received prior instruction as to conducting a patient interview nor formulating a SOAP note, but instead received feedback as the course progressed on how to improve upon skills. Each course was a two-credit course and consisted of four patient cases per semester that took approximately four class sessions to complete. The two-semester case study course did not formally integrate the PPCP though the various components that were a part of the process. The instructors did not specifically lead students through the incorporation of the PPCP. Following the inclusion of the PPCP within the United States ACPE accreditation standards, the faculty sought to redesign the curriculum to more formally incorporate the process within the curriculum. In lieu of the original two-semester problem-based course, PharmD students would participate in a new four-semester course series longitudinally throughout pharmacy years two and three (PY2, PY3). Each course was worth two credit hours with the exception of the fourth course in the series, which was worth three credit hours. Students are introduced to the PPCP during pharmacy year one (PY1). Beginning in the fall of PY2, the first course in the new four-semester case-based series is the students’ first opportunity to apply the PPCP. During the first (fall) semester of the PY2 year, the collect and assess steps are reviewed in depth, while the plan and implement steps are introduced. Students then work in small groups on patient cases utilizing process-oriented guided inquiry learning (POGIL) and are then individually assessed on their ability to implement these PPCP components through an essay. Students work through multiple patient cases throughout the semester that correlate with students’ previous and current pharmacotherapy courses. In the spring PY2 semester, the same steps are further reinforced via problem-based patient cases that increase in complexity and are completed in small group settings. Additionally, the defense of students’ case plans and patient counseling practice is incorporated into the course. The intent is to reinforce PharmD students’ understanding of each component of the process while increasing critical thinking and problem-solving abilities. Throughout the PY3 fall and spring semesters, students are expected to implement all five components of the PPCP as they work through case-based problems each week, defend their care plans, and practice counseling patients on medications. They create follow-up care plans as part of their plan. Activities in the fall of PY3 occur via small groups, while course assignments during the spring of PY3 occur individually in order to ensure the ability to autonomously create, implement, and defend a care plan. In regard to the PPCP, the redesigned course series was developed to provide PharmD students with a foundation for implementing the PPCP via a stepwise approach while facilitating intentional repetition throughout PY2 and PY3. Prior to this redesign, PharmD students participated in a two-semester problem-based case study course during PY2. While the two-semester course integrated the PPCP, the emphasis was on solving the problem with no formal instruction nor objectives tied directly to the PPCP. The researchers hypothesized that the redesigned course would improve students’ perceived ability to implement the PPCP.

## 2. Materials and Methods

PharmD students completing the original case study series (two semesters) during their PY2 year and students completing the first two semesters of the redesigned 4-semester course during PY2 were given a voluntary 12-item survey administered electronically at the beginning of the PY3 fall semester for the respective cohort after completion of the case study courses during the prior PY2 to assess their perceived ability to integrate the PPCP into patient care. Since the first 2 semesters of the redesigned course focused on the first 4 steps of the PPCP, there were no survey items pertaining to monitoring and follow-up, which fall under PPCP step 5. Students were, however, asked to rate their perceived ability to ask appropriate questions during a patient interview, identify medication-related problems (MRPs), and document both medication-related problems and patient interactions since this is important to both the PPCP and is an outcome of the case study course series. The survey was given to both student cohorts at the beginning of the fall PY3 semester after the cohorts had completed two semesters of the original and redesigned case study course. The estimated time to complete the survey was five minutes. The survey included baseline demographics, as well as questions assessing the students’ perceived ability to integrate the PPCP and the perceived importance of each aspect of the PPCP to pharmacist-delivered patient-centered care. Survey items pertaining to the students’ perceived abilities were assessed via a continuous 10-point scale, with ten being an excellent or very high perceived ability to complete the task and zero being no perceived ability to complete the task. Questions related to the students’ perceived importance of asking pertinent questions, identifying MRPs, and documentation were assessed via a 5-point Likert scale. All items were developed by the researchers utilizing the PPCP domain descriptions as the framework [1]. Prior to administration, the survey was reviewed by pharmacy practice faculty from the College of Pharmacy working in various practice settings to identify survey items that were not clear. The researchers adjusted survey items based on the feedback provided. The primary outcome of the study was the students’ perceived ability to implement the applicable PPCP steps after completing the second course of the new series compared to perceptions of students completing the original, 2-semester case study course prior to being restructured. Secondary outcomes included students’ perceived importance of asking pertinent questions, identifying MRPs, and documentation. Student letter grades in the course are provided for informational purposes only and were not an outcome of the study. Authors reviewed grades but from an observational standpoint only. Statistical analyses were conducted using Python 3.10.12 (Python Software Foundation, Scotts Valley, CA, USA). Reliability of the survey instrument was measured using Cronbach alpha, with a coefficient of >0.70 indicating good internal consistency. Student perceptions were analyzed using the nonparametric Mann–Whitney U test. A summated scaled score was computed for each student to capture perceived abilities overall. Chi-square test was used to examine differences in demographic characteristics between groups. Levels of significance were tested at *p* < 0.05. This study was approved by the University Institutional Review Board and informed consent was obtained from all participants. The survey was administered electronically via SurveyMonkey^®^. Participant identities were blinded to the investigators. 

## 3. Results

One hundred seven PharmD students completed the survey, including 61 students (response rate of 80.2%) from the original two-semester case study series and 46 students (response rate of 74.2%) from the four-semester redesigned course. The majority of the students completing the survey were female and had experience as a pharmacy technician in the community setting (Table 1). The 12-item survey was found to be reliable, with a Cronbach alpha coefficient of 0.825. The items from the survey are listed in Table 2 and Table 3. There were no differences between the groups in terms of the baseline demographics. The average score for each question asking students to rate their ability is listed in Table 2. The remaining questions (Table 3) asked students to identify their level of agreement with statements related to the integration of the PPCP into practice. The percent of students agreeing or strongly agreeing with these statements is provided in Table 3. The results of the nonparametric Mann–Whitney U test suggested that students completing the redesigned case study series had significantly higher perceptions of their ability to integrate the PPCP compared to the students completing the original series (*p* < 0.001) (Table 2). A statistical difference was seen for items 1 through 5, and item 8. For items 6 and 7, no statistical difference was identified (Table 2). The students’ perception of the importance of asking pertinent questions, identifying MRPs, and documentation in patient care did not differ between the cohorts (Table 3). The letter grades of the students completing the original two-semester course for course 2 included 51 As, 20 Bs, and 1 C. For those students completing the redesigned course series, at the conclusion of the second course, the letter grades were 38 As, 23 Bs, and 3 Cs.

## 4. Discussion

A significant difference in the students’ perceived ability to implement the PPCP after completing just two semesters of the redesigned course series versus students who completed the original two-semester course was observed in this study. When examining each item, the students perceived the greatest increase in their ability to collect, assess, and plan. This could be indicative of the fact that the restructured course builds on each component of the PPCP and allows for several opportunities to implement these components into practice in a classroom setting in a more guided fashion during the first two semesters. While all the steps are covered, a strong emphasis in semesters one and two of the course is on the collect and assess steps. Since the students are briefly introduced to documentation during the PY2 fall semester and produce one note per group per case as documentation during the PY2 spring semester, it is possible they perceived a lower ability to document since they have not been asked to do so individually at this point in the course. This aspect of the course design was consistent between the original and redesigned cohorts. 

Of the available studies, often, PPCP integration occurs at the end of the didactic curriculum [3,9]. Our study employed PPCP integration longitudinally across PY2 and PY3, allowing for more opportunities for repetition. Gonyeau et al. integrated the PPCP into a disease management course similarly across PY2 and PY3 in which the implementation methods were not standardized; however, the PPCP was a small portion of the course as the course covered patient assessment, pathophysiology, self-care, and therapeutics [8]. They also assessed faculty perceptions of integration and assessed student understanding through various means including multiple choice questions, homework assignments, and concept maps [8]. The implementation described in the current study offers a standardized means for integration of the PPCP in which the implementation of the PPCP is a main component of the course. Alsharif et al. trialed integration into a medicinal chemistry course; however, the integration emphasized knowing what the process is and how it can be integrated [7]. Our study examined students’ perceptions of their ability to integrate the process, not just understand what it is. Similar to our study, Nasser et al. examined students’ perceptions of their preparedness to apply the PPCP [4]. The PPCP was integrated into a laboratory course during PY2. Similar to our study, they noted an improved perception of students’ preparedness to collect pertinent patient information and to develop an individual patient-centered care plan [4]. The students in the current study identified an improved perception of their ability to ask appropriate questions, and identify and solve MRPs. In the current study, there was no significant difference in the students’ perception of the importance of asking appropriate questions, identifying MRPs, and documentation in actual practice between groups, with most students finding each aspect very important. This may be indicative of the students’ perceived clinical relevance, which can play a role in retention [10]. Noureldin et al. evaluated PY3 students’ perceived self-efficacy for implementing the PPCP before and after completing a case-based capstone course and noted a significant difference in the students’ self-efficacy related to applying the collect, assess, and plan portions of the PPCP when comparing before and after course completion [3]. 

There are several limitations to this study. The overall student cohort was small, with 61 students from the original case study series and 46 students from the redesigned course participating in the survey. The survey was conducted after the students completed just two semesters of the redesigned course to mirror the time of the students completing the original two-semester series. Therefore, the full effect of the redesigned course on student perceptions after completion of the full, four-semester series is not known. Additionally, the actual effect on student performance remains unknown. The student performance remained the same overall, as evidenced by the course grades. Though the students noted a perceived improvement in ability, their actual ability to implement the process remains untested. Nasser et al. noted the students’ perceptions of preparedness persisted throughout an introductory pharmacy practice experience, but data regarding perception and actual ability are lacking [4]. Further research in this area will help determine how these perceptions translate to APPE performance. While evaluating student perception has its limitations, it is important that students develop the ability to self-assess, a skill which is a critical component of continuous professional development [11]. Future studies should evaluate student performance on assessments before and after integration of the PPCP into a course. Additionally, the impact of integrating the PPCP during didactic semesters on students’ performance during APPEs would provide further insight into the impact of didactic integration. Further, this knowledge would begin to deepen our understanding of where and how the PPCP should be integrated in order to achieve student implementation during APPEs and in practice. 

## 5. Conclusions

The implementation of a structured approach to the PPCP in a redesigned four-semester case-based course resulted in an increase in pharmacy students’ perception of their ability to implement the process in patient care. Further study is needed to determine if students’ perception of their ability to implement the PPCP into practice correlates with their actual ability to do so. 

## Figures and Tables

**Table 1:** Baseline demographics.

	Original Course (n = 61)	Redesigned Course (n = 46)	*p*-Value
Gender			
- Male	22 (36%)	21 (46%)	0.32
Age			
- 21–29	48 (79%)	41 (89%)	0.15
- ≥30	13 (21%)	5 (11%)	0.15
Pharmacy technician experience			
- No experience	26 (43%)	20 (43%)	0.93
- <1 year	3 (5%)	5 (11%)	0.25
- 1–5 years	24 (39%)	18 (39%)	0.98
- >5 years	8 (13%)	3 (7%)	0.27
Pharmacy technician setting	n = 35	n = 26	
- Community	30 (85%)	22 (85%)	0.80
- Other	5 (15%)	4 (15%)	0.90
Highest degree			
- Associate	20 (33%)	9 (20%)	0.13
- Bachelor’s	35 (57%)	33 (72%)	0.13
- Master’s/Doctorate	6 (10%)	4 (8%)	0.84

**Table 2:** Pharmacy students’ perceived ability to implement components of the patient care process before and after completing a redesigned course series.

Statement: Please Rate Your Ability to…	Original Course Series Mean ± SD * (n = 61)	Redesigned Course Series (n = 46) Mean ± SD	
Asking all pertinent or needed questions during a patient interview	6.4 ± 1.7	8.2 ± 1.2	<0.001
Asking questions in an appropriate manner for interaction with patients (patient specific language, appropriate order and follow-up questions, etc.)	6.9 ± 1.5	8.6 ± 1.0	<0.001
Identifying medication related problems for a specific patient	6.3 ±1.6	7.5 ± 1.5	<0.001
Documenting medication related problems for a specific patient	6.7 ± 1.8	7.7 ± 1.5	<0.001
Determining a method for managing or solving medication related problems	6.5 ± 1.7	7.6 ± 1.4	<0.001
Ensuring care provided is patient-centered	7.8 ± 1.7	8.3 ± 1.4	0.184
Documenting patient interactions in an organized format (example SOAP)	7.6 ± 1.8	7.5 ± 1.8	0.733
Solve medication related problems for my patients	6.3 ± 1.9	7.5 ± 1.5	0.001

* SD: standard deviation.

**Table 3:** Students’ perceived importance of applying PPCP to practice after completion of redesigned versus original case study series.

Statement:	Original Course Series (% * Strongly Agree, Agree) n = 61	Redesigned Course Series (% Strongly Agree, Agree) n = 46
I believe being able to ask all appropriate questions during a patient interview is an important skill to possess as a pharmacist	95.2	95.7
I believe being able to identify medication related problems is an important skill to possess as a pharmacist	95.3	95.6
I believe being able to document medication related problems appropriately is an important skill to possess as a pharmacist	92.1	93.5
I believe documenting patient interactions in an organized format is an important skill to possess as a pharmacist	90.5	86.9

* %, percent; *p* > 0.05.

## Data Availability

The data presented in this study are available on request from the corresponding author. The data are not publicly available due to privacy restrictions.
